# Standardized cardiovascular magnetic resonance imaging (CMR) protocols, society for cardiovascular magnetic resonance: board of trustees task force on standardized protocols

**DOI:** 10.1186/1532-429X-10-35

**Published:** 2008-07-07

**Authors:** Christopher M Kramer, Jorg Barkhausen, Scott D Flamm, Raymond J Kim, Eike Nagel

**Affiliations:** 1 Departments of Medicine and Radiology, University of Virginia Health System, Charlottesville, VA, USA; 2 Clinic for Radiology and Nuclear Medicine, University Hospital, Lubeck, Germany; 3 Imaging Institute, Cleveland Clinic, Cleveland, OH, USA; 4 Duke Cardiovascular Magnetic Resonance Center, Duke University Medical Center, Durham, NC, USA; 5 Division of Imaging Sciences, King's College, London, UK

## Abstract

**Index:**

1. General techniques

1.1. Stress and safety equipment

1.2. Left ventricular (LV) structure and function module

1.3. Right ventricular (RV) structure and function module

1.4. Gadolinium dosing module.

1.5. First pass perfusion

1.6. Late gadolinium enhancement (LGE)

2. Disease specific protocols

2.1. Ischemic heart disease

2.1.1. Acute myocardial infarction (MI)

2.1.2. Chronic ischemic heart disease and viability

2.1.3. Dobutamine stress

2.1.4. Adenosine stress perfusion

2.2. Angiography:

2.2.1. Peripheral magnetic resonance angiography (MRA)

2.2.2. Thoracic MRA

2.2.3. Anomalous coronary arteries

2.2.4. Pulmonary vein evaluation

2.3. Other

2.3.1. Non-ischemic cardiomyopathy

2.3.2. Arrhythmogenic right ventricular cardiomyopathy (ARVC)

2.3.3. Congenital heart disease

2.3.4. Valvular heart disease

2.3.5. Pericardial disease

2.3.6. Masses

## 1. General Techniques

### 1.1. Stress equipment and safety

#### Equipment

1. Monitoring equipment (blood pressure, electrocardiogram for monitoring of cardiac rhythm, intercom to communicate with patient)

2. Preparation and regular practice for rapid removal of the patient from the magnet

3. Emergency resuscitation policy in place

4. Defibrillator

5. Drugs for emergency treatment

a. Immediately at hand: β-blocker (eg esmolol or metoprolol), nitroglycerin, aminophylline

b. In the emergency cart: full set of emergency drugs (including drugs such as: epinephrine, beta blockers, atropine, bronchodilators, antiarrhythmic drugs)

6. For dobutamine – on-line assessment of wall motion during image reconstruction performed immediately after image acquisition

#### Stress agents

Dobutamine typical maximum dose of 40 ug/kg/min

Atropine 0.25 mg fractions typical (maximal dose 2 mg)

Adenosine 140 μg/kg body weight/min

#### Contraindications

##### Dobutamine

• Severe systemic arterial hypertension (≥ 220/120 mmHg)

• Unstable angina pectoris

• Significant aortic valve stenosis (Peak aortic valve gradient > 50 mmHg or aortic valve area < 1 cm^2^)

• Complex cardiac arrhythmias including uncontrolled atrial fibrillation

• Hypertrophic obstructive cardiomyopathy

• Myocarditis, endocarditis, pericarditis

• Uncontrolled congestive heart failure

##### Adenosine

• Known hypersensitivity to adenosine

• Known or suspected bronchoconstrictive or bronchospastic disease

• 2^nd ^or 3^rd ^degree atrioventricular (AV) block

• Sinus bradycardia (heart rate < 45 bpm)

• Systemic arterial hypotension (< 90 mmHg)

##### Atropine

• Narrow-angle glaucoma

• Myasthenia gravis

• Obstructive uropathy

• Obstructive gastrointestinal disorders

#### Patient preparation

1. Obtain informed consent for the stress test

2. To fully exert its effects patients should refrain from the following medications for at least 24 hours prior to the examination due to potential of counteraction against stress agent:

Dobutamine: β-blockers and nitrates

Adenosine: caffeine (coffee, tee, caffeinated beverages or foods e.g. chocolate, caffeinated medications), theophylline, dipyridamole

3. Fasting is not usually considered mandatory, but is sometimes advised because recognized adverse effects of stress drugs include nausea and vomiting, which is problematic when lying supine in the restricted space of the magnet

#### Potential adverse effects

Dobutamine at high doses may cause chest pain, palpitations. More severe complications are uncommon, including:

• infarction

• ventricular fibrillation

• sustained ventricular tachycardia

Adenosine may cause flushing, chest pain, palpitations, breathlessness. More severe side effects include

• transient heart block

• transient hypotension

• transient sinus tachycardia

• bronchospasm

### 1.2. Left ventricular structure and function module

1. Scout imaging – transtransaxial, coronal, sagittal

2. Transaxial (8–10 mm) set of steady state free precession (SSFP) or fast spin echo images through the chest.

3. Scout to line up short axis images – cine acquisitions are preferable to single shot as long axis motion and inflow should be visualized

a. Vertical long axis prescribed orthogonal to transaxial scouts aligned through the apex and center of the mitral valve

b. Horizontal long axis aligned orthogonal to the vertical long axis, passing through the apex and center of the mitral valve

4. Steady state free precession short axis cine images, from the mitral valve plane through the apex. The basal most short axis slice should be located immediately on the myocardial side of the atrioventricular junction at end-diastole prescribed from the previously acquired long axis cines.

a. Slice thickness 6–8 mm, with 2–4 mm interslice gaps to equal 10 mm.

b. Temporal resolution ≤ 45 ms between phases

c. Parallel imaging used as available

5. Steady state free precession long axis cine images

a. The 4 chamber long axis is prescribed from the vertical long axis through the apex and center of the mitral and tricuspid valves. This can be cross-checked on basal short axis cines, using the costophrenic angle (margin) of the RV free wall.

b. Vertical long axis, prescribed from the scout already acquired

c. LV outflow tract (LVOT) long axis, passing through the apex, the center of the mitral valve and aligned with the center of LVOT to aortic valve, as seen on a basal short axis cine.

d. Optional – a set of more than 3 rotational long axis views can be obtained.

6. Analysis

a. All short axis images are evaluated with computer-aided analysis packages for planimetry of endocardial and epicardial borders at end-diastole and end-systole. More advanced software automatically adjusts for systolic atrioventricular ring descent.

b. The inclusion or exclusion of papillary muscles in the LV mass should be the same as that used in normal reference ranges used for comparison.

c. Care must be used at the 1 or 2 most basal slices. Due to systolic movement of the base towards the apex in normally contractile ventricles, the end-systolic phase will include only left atrium. This may not be the case in a severely dysfunctional LV. Either way, this slice at end-diastole will include LV mass and volume.

### 1.3. Right ventricular structure and function

1. Right ventricular (RV) short axis views can be obtained in a similar fashion to the LV structure and function module. If the short axis is used for quantification, it is important to place the basal short axis slice immediately on the myocardial side of the right ventricle and to take extra care to exclude appropriate amounts of atrial volume from at least one basal slice at end systole.

2. Transaxial stack of cines covering the RV enable best identification of the tricuspid valve plane.

3. Long axis images should include an RV vertical long axis view aligned with tricuspid inflow and a RV outflow tract view (sagittal plane through the pulmonary valve).

4. Analysis

a. A similar computer-aided analytic approach is required as for the left ventricle.

b. Care must be taken with RV trabeculations and with the RV outflow tract after repair of tetralogy of Fallot with a consistent approach used for longitudinal comparison.

### 1.4. Gadolinium dosing module

Notes:

1. Volumes and injection rates depend on scan duration: the given values are recommendations for typical scan times.

2. Injection rates are different for 1 molar contrast agents. As a general rule, divide the given injection rates by a factor of 2.

3. Contrast agents with higher relaxivity (e.g., gadobenate dimeglumine) require smaller doses.

4. Throughout the protocols, the term "gadolinium" refers to gadolinium chelates

5. Injection rate for peripheral angiography with elliptic-centric readouts may be different than those specified below.

See Table 1.

**Table 1:** Contrast and chasing bolus doses and injection rates

**Indication**	**contrast dose ** (mmol/kg body weight unless otherwise stated)	**injection rate**	**chasing ** **bolus** ** (NaCl)**	**injection rate**
**Perfusion**	0.05–0.1	3–7 ml/s	30 ml	3–7 ml/s
**Late gadolinium ** **enhancement**	0.1–0.2		20 ml	
**Angiography ** **(carotids, renals, thoracic or ** **abdominal Aorta)**	0.1–0.2	2–3 ml/s	20 ml	2–3 ml/s
**Time-resolved ** **angiography**	10 ml	3–5 ml/s	30 ml	4 ml/s
**Peripheral angiography**	0.2 mmol/kg	first 10 ml @ 1.5 ml/s, rest @ 0.4–0.8 ml/s	20 ml	0.4–0.8 ml/s

### 1.5. First pass perfusion module

1. Scout imaging as per LV structure and function module

2. Saturation-recovery imaging with gradient echo-echo planar (GRE-EPI) hybrid, GRE, or SSFP readout

3. Short-axis view imaging (at least 3 slices per heart beat)

a. For ischemia evaluation, must obtain data every heart beat

b. Slice thickness 8 mm

c. Parallel imaging, 2-fold acceleration, if available

d. In-plane resolution, ~< 3 mm

e. Readout temporal resolution ~100 – 125 ms or shorter as available

f. Contrast is given (0.05 – 0.1 mmol/kg, 3–7 ml/s) followed by at least 30 ml saline flush (3–7 ml/sec)

g. Breathhold starts during early phases of contrast infusion before contrast reaches the LV cavity.

h. Image for 40–50 heart beats by which time contrast has passed through the LV myocardium

### 1.6. Late gadolinium enhancement module

1. Need at least 10 minute wait after gadolinium injection (0.1–0.2 mmol/kg). Note – The delay may be shorter than 10 minutes if lower doses are used as blood pool signal falls below that of late enhanced myocardium.

2. 2D segmented inversion recovery GRE imaging during diastolic stand-still

3. Same views as for cine imaging (short- and long-axis views)

4. Slice thickness, same as for cine imaging

5. In-plane resolution, ~1.4–1.8 mm

6. Acquisition duration per R-R interval below 200 ms but should be less in the setting of tachycardia.

7. Inversion time set to null normal myocardium. Alternative is to use fixed TI with a phase-sensitive sequence.

8. Read-out is usually every other heart beat but should be modified to every heart beat in the setting of bradycardia, and every third heart beat in the setting of tachycardia or arrhythmia.

9. Optional

a. Single-shot imaging (SSFP readout) performed as backup for patients with irregular heart beat, difficulty breath holding.

b. 3D sequences with parallel imaging in appropriate patients if signal-to-noise is sufficient.

10. Analysis

a. Interpret visually using AHA 17-segment model.

b. Estimate area (mean transmural extent) of enhancement within each segment (0%, 1–25%, 26–50%, 51–75%, 76–100%).

## 2. Disease specific protocols -

### 2.1. Ischemic heart disease

#### 2.1.1. Acute myocardial infarction

1. LV structure and function module

2. Optional – T2 weighted imaging (see nonischemic cardiomyopathies protocol for sequence details) (at least areas with wall motion abnormalities)

3. First pass perfusion module (only at rest)

4. To look for microvascular obstruction, scan for consider repeat first pass perfusion sequence or early gadolinium enhancement, i.e within the first 1–3 minutes after contrast infusion

5. Late gadolinium enhancement module

#### 2.1.2. Chronic ischemic heart disease and viability

1. LV structure and function module

2. Optional – low dose dobutamine with 5–10 minute infusion of 10 μg/kg/min of dobutamine to assess contractile reserve as improvement in wall thickening

3. Optional – adenosine stress-rest perfusion or high dose dobutamine functional imaging (see stress protocols for more details) to determine the presence of inducible perfusion deficits or wall motion abnormalities

4. Late Gadolinium Enhancement module

5. Analysis

a. Helpful to view cines and LGE images of equivalent planes side-by-side

b. Interpret using both cine and LGE data. For example, "region is dysfunctional but viable".

#### 2.1.3. Dobutamine stress CMR

1. LV structure and function module

2. Dobutamine stimulation

a. Increase the dobutamine in increments of 10 μg/kg body weight/minute every 3 minutes starting at 10 μg/kg body weight/minute until target heart rate [85% × (220-age)] reached.

b. Add atropine in small incremental doses, if heart rate response is poor.

c. Repeat 3 short axis and 3 long axis cine views during each increment

d. Continuous ECG monitoring and BP measured during each stage.

e. View cine loops online as they are being acquired.

f. Adapt the SSFP cine sequence to optimize temporal resolution as needed as the heart rate increases.

g. Stop test for new wall motion abnormality, serious side effect, or achievement of peak heart rate.

3. Analysis

a. View cines in multiscreen format, reviewing rest, intermediate stress levels and peak stress at the same time in a synchronized fashion.

b. Describe wall motion as normokinetic, mild hypokinetic, severe hypokinetic, akinetic and dyskinetic for all 17 segments.

c. Report inducible wall motion abnormalities and viability.

#### 2.1.4. Adenosine stress perfusion CMR

1. LV structure and function module (alternatively this can be performed between stress and rest perfusion, although performance immediately after gadolinium infusion may reduce the contrast of the blood-endocardium interface)

2. Two intravenous lines should be available, one for gadolinium and one for adenosine, one in each arm. Preferential site of contrast infusion is antecubital. Blood pressure cuff should be used with care taken not to interfere with gadolinium or adenosine infusion.

3. Adenosine stress perfusion imaging (at least 3 minute infusion of 140 ug/kg body weight/min). Option – initial adenosine infusion may be performed with the patient outside the bore of the magnet.

a. First pass perfusion module

b. During last minute of adenosine, gadolinium is injected

c. After imaging for 40–50 heart beats by which time gadolinium has passed through the LV myocardium, adenosine is stopped.

d. Continous ECG monitoring and BP measured at baseline, during infusion, and for at least 2 minutes post-infusion of adenosine.

4. Rest Perfusion

a. Need at least 10 minute wait for gadolinium to wash out from stress perfusion imaging. During this period stress images can be reviewed, cine imaging can be completed (e.g long-axis views), valvular evaluation can be performed, etc.

b. Perfusion imaging repeated without adenosine using same dose of gadolinium

c. If stress images are normal and free of artifacts, rest perfusion can be skipped. Additional gadolinium may be given as needed for late gadolinium enhancement (for a total of 0.1 – 0.2 mmol/kg)

5. Late Gadolinium Enhancement module

a. Need to wait at least 5 minutes after rest perfusion

6. Analysis

a. Interpret visually using 17 segment AHA segment model (16-segment model can be used, leaving out apex)

b. Optional – Quantitative analysis of the inflow curves could be considered in cases without obvious visual perfusion defect.

c. Helpful to view cines, stress and rest perfusion, and LGE images all side-by-side in equivalent slices

### 2.2. Angiography

#### 2.2.1. Peripheral MRA

1. Peripheral vascular coil, or combination of coils, as available. Venous compression cuffs (placed on the thighs, and inflated to sub-diastolic pressure) are helpful, if available.

2. Transaxial, low-resolution, vessel scouting with time-of-flight MRA or SSFP.

3. Gadolinium timing

a. Option 1 -Transaxial test bolus at level of distal abdominal aorta. 2 ml injection of gadolinium, followed by 20 ml saline. Determine time to peak enhancement following injection using a single-shot bolus tracking sequence.

b. Option 2 – Bolus trigger technique to time start of scan

4. Stepping-table, gadolinium-enhanced MRA performed in the coronal projection from the mid abdominal aorta to the feet.

a. Two volumetric acquisitions – one pre-contrast (for subtraction) and one during contrast administration.

b. Gadolinium injected in 2 phases to minimize venous contamination followed by saline bolus.

c. Slice thickness 1–1.5 mm; acquired spatial resolution in-plane 0.8–1.5 mm.

d. Slices – typically 60–80, as needed to accommodate vessels of interest.

e. Volumes obtained of abdomen/pelvis and thighs may be coarser spatial resolution (larger vessels), while those of the legs preferably are sub-millimeter spatial resolution. The former acquisitions typically require 15–20 seconds, while the leg acquisition may take 60–90 seconds for increased spatial resolution. Elliptical centric k-space acquisition is advantageous for the legs. If available, time-resolved acquisitions are preferred for the legs.

f. Parallel acquisition recommended (multichannel surface coil needed)

5. Analysis

a. 3D reconstructions may be helpful for an initial overview and visualizing the vasculature tree, but generally should not be used for primary decision making.

b. Primary diagnoses are made by scrolling through source images (typically coronal and/or sagittal), and using selected thin slab MIP and MPR reconstructions in optimized orthogonal and oblique views for each station. The presence, number, and degree of stenoses are evaluated qualitatively.

Alternative: dual injection protocol

1. Single dose of gadolinium: time-resolved MRA of the calf and foot vessels

2. Single dose of gadolinium: abdominal and thigh vessels

#### 2.2.2. Thoracic aortic MRA

1. Localizer, 3 orientations

2. Half-fourier single shot fast spin echo or SSFP (one breathhold, entire thorax)

Transaxial orientation.

3. Transaxial T1-weighted fast spin echo through aorta (for intramural hematoma, dissection)

4. SSFP cine imaging in parasagittal plane parallel to aorta Option – use 3-point piloting

5. Evaluate aortic valve as per valvular protocol

6. Contrast timing

a. Option 1 -Transaxial test bolus at level of distal abdominal aorta. 2 ml injection of gadolinium, followed by 20 ml saline. Determine time to peak enhancement following injection.

b. Option 2 – Bolus triggering technique to time start of scan

c. Option 3 – Rapid multiphase 3D acquisitions without timing

7. 3D gadolinium enhanced MRA (0.1–0.2 mmol/kg) (optional – ECG-gated acquisition)a. Use spatial resolution of at least 1–1.5 mm b. Parallel acquisition if availablec. At least 2 acquisitions after contrast injection

8. Optional – transaxial T2-weighted gradient echo or T1-weighted gradient-echo post-contrast for aortitis

9. Analysis – MPR-Reconstruction, MIP and thin slab MIP

#### 2.2.3. Anomalous coronary artery evaluation

1. LV structure and function module to look for wall motion abnormalities

a. Add repeat horizontal long axis with high temporal resolution sequence (≤ 20 ms per phase) to accurately determine quiescent period of RCA

2. Navigator-gated, 3D, free-breathing, MRA sequence:

a. Transaxial slices spanning from level of proximal main pulmonary artery down to the middle of the right atrium (entire cardiac coverage if desired). Slice thickness 1–1.5 mm; acquired spatial resolution in-plane of 1.0 mm or less.

b. Slices – typically 50–80, as needed to encompass vessels of interest.

c. Adjust trigger delay and acquisition window according to observed quiescent coronary period.

d. Parallel acquisition preferred

e. Navigator placed over the right hemi-diaphragm.

f. Optional – contrast to increase vessel conspicuity

3. Optional -

a. breathhold techniques if poor image quality or navigators unavailable or of poor quality

b. T2-prepared sequence may be useful

#### 2.3.4. Pulmonary vein evaluation – pre- and post-ablation

1. LV structure and function module

2. Breathhold non-gated contrast-enhanced MRA performed in the coronal projection encompassing the pulmonary veins and left atrium (greater anterior coverage if breathholding permits) (optional – optimize oblique projections, ECG-gated acquisition)

a. Gadolinium (0.1–0.2 mmol/kg) injected at 2–3 ml/s

b. Slice thickness 1–2 mm; acquired spatial resolution in-plane 1–1.5 mm.

c. Slices – typically 60–80, as needed to encompass region of interest.

d. Parallel acquisition used as available

e. 2–3 volumetric acquisitions – each breathhold typically no longer than 15–18s.

3. Optional – through plane phase contrast flow analysis through each pulmonary vein.

4. Analysis

Gadolinium MRA evaluated qualitatively by scrolling through slices in various orientations. The number and position of pulmonary veins is accounted for noting common trunks, accessory veins, and evidence for stenosis or thrombosis. A 3D workstation is used for MPR analysis to calculate major and minor axes, and cross sectional area of each pulmonary vein ostium. Compare pre- and post-ablation images side by side. 3D rendering may facilitate planning of surgical or electrophysiologic interventions.

### 2.3. Other

#### 2.3.1. Nonischemic LV cardiomyopathies including myocarditis

1. LV structure and function module

2. Consider T2-weighted imaging in the acute setting when necrosis/edema may be present (e.g. myocarditis)

a. Body coil should be used or alternatively functional surface coil intensity correction algorithms

b. Breath-hold, segmented fast spin-echo imaging (double inversion recovery)

c. Perform imaging prior to contrast administration

d. Selected slices based on cine imaging findings (e.g. 2 and 4-chamber long axis and 3 representative short axis slices)

e. Adjust readout to mid-diastole

f. Slice thickness at least 10 mm

g. Slice thickness of dark blood prep if used should be greater than the base-apex motion of the mitral annulus

3. Late Gadolinium Enhancement module

a. Analysis – Examine the "pattern" of enhancement as certain nonischemic cardiomyopathies have predilection for scarring in typical ways

4. Optional – adenosine stress-rest perfusion imaging or high dose dobutamine stress functional imaging (see stress protocols) to comment on the presence or absence of ischemia since a mixed cardiomyopathy may be present

5. For hypertrophic cardiomyopathies -LV outflow tract flow imaging for presence of turbulence, systolic anterior motion, and phase velocity measures of gradient if present.

#### 2.3.2. Arrhythmogenic right ventricular cardiomyopathy

1. LV structure and function module – consider 5–6 mm slice thickness

a. Careful RV as well as LV volumetric analysis.

2. Transaxial or oblique transaxial SSFP cine images covering the RV including RVOT. An RV vertical long axis view aligned with tricuspid inflow is recommended.

3. Optional sequences

a. Selected transaxial or oblique transaxial black blood images (double inversion recovery T1-weighted fast spin echo).

b. Repeat same geometry with fat suppression

c. Late gadolinium enhancement module in same orientations as above. Consider T1 nulling for RV.

d. Consider use of anterior surface coil only to improve resolution without "wrap around" artifacts.

e. Consider prone position in overweight patients in order to minimize distance between the surface coil and RV.

Major criteria for diagnosing ARVC include severe dilation and reduction of right ventricular ejection fraction with no or mild left ventricular involvement; localized right ventricular aneurysms (akinetic or dyskinetic areas with diastolic bulgings); severe segmental dilation of right ventricle. Minor criteria include mild global right ventricular dilation or ejection fraction reduction with normal left ventricle; mild segmental dilation of right ventricle; regional right ventricular hypokinesia. All of these are demonstrable by CMR as performed above. CMR also allows assessment of fatty infiltration and myocardial fibrosis. However, the latter two findings are not part of current guidelines.

Note – there is variability of the structure and shape of the RV amongst normals, so there is a tendency for inexperienced observers to overdiagnose RV wall motion abnormalities. For example, relative end-systolic bulging of a thin but contractile region of the RV free wall adjacent to the moderator band can be a normal finding and basal short axis cines may give the impression of inferior wall dyskinesis due to normal through-plane motion. Careful study of RV cines in volunteers and a range of patients is recommended before attempting identification of ARVC.

#### 2.3.3. Congenital Heart Disease

For all diagnoses:

1. LV and RV structure and function module

a. In many cases, a contiguous stack of transaxial SSFP cine images from the inferior wall of the LV through the top of the aortic arch is recommended.

b. Gradient echo cine or hybrid gradient echo/echo planar imaging may be added for improved detection of turbulent flow in particular planes

2. Scout imaging in the plane of the ascending aorta and main pulmonary artery.

3. Through plane velocity encoded cine imaging through the main pulmonary artery and aorta to measure Qp and Qs.

4. Time resolved or multiple rapid dynamic 3D gadolinium enhanced magnetic resonance angiography in a coronal orientation

For individual diagnoses, protocols must be individualized. Additional sequences to consider:

1. For shunt lesions

a. Measurement of Qp:Qs as above

b. In plane and/or through plane velocity encoded gradient echo sequence to visualize rather than measure shunt flow

2. For lesions involving the great vessels

a. SSFP cine imaging in parasagittal plane parallel to aorta

b. Specific alignment of cines with jet flow through aortic coaractation, flowed by through-plane jet velocity mapping to measure peak velocity and recognize possible diagnostic prolongation of forward flow

c. Assessment of valves as per valvular protocol

d. SSFP cine and/or gradient echo cine images along the individual pulmonary arteries

e. Comparison of RPA with LPA flow volume in the case of unilateral branch PA stenosis.

#### 2.3.4. Valvular disease

Patients with artificial valves can safely undergo CMR at 1.5 and 3 Tesla. The force exerted by the beating heart is many-fold higher than the force exerted by the magnetic field.

1. LV structure and function module.

a. Use horizontal long axis to look for valve anatomy and turbulence of the mitral and tricuspid valve.

b. Use LVOT view for mitral and aortic valve.

c. Use vertical long axis for mitral valve.

d. Coronal view for aortic valve

e. Additional views (RV long axis, RV-outflow tract as needed).

2. Specific

a. Valve morphology assessment with SSFP cine in the plane of the valve in question. Care must be taken to optimize the level and angle of imaging

b. Note – if planimetry of a stenotic valve is to be attempted, a contiguous or slightly overlapping stack of high resolution cines transecting the line of the jet and moving from orifice level to immediately downstream is recommended. Planimetry is most likely to be valid where the cross section of the orifice, or rather of the jet, is clearly delineated. This may not always be the case due to fragmented or oblique jet flow.

c. Gradient echo or hybrid gradient echo/echo planar imaging may visualize regurgitant jets with a higher sensitivity (for qualitative purposes only).

d. In mitral or tricuspid regurgitation, a contiguous stack of 5 mm cines is recommended aligned with the direction of inflow and transecting the principal line of coaptation, moving from the more superior commissure to the inferior. The orientation can be that of the LVOT plane for the mitral and transaxial for the tricuspid. Such a stack enables assessment of tethering, prolapse, or regurgitation through the scallops of both mitral leafletes.

3. Measure flow velocity and volume perpendicular to the vessel distal to valve leaflet tips.

a. Adapt velocity encoding to actual velocity (using lowest velocity without aliasing).

b. Use lowest TE possible for high velocity jet flows.

c. The CMR software should routinely correct for phase errors due to concomitant gradients. As long as this is the case, background phase errors due to eddy currents may be correctable by normalizing velocities to reference in static tissue.

4. Analysis:

a. Determine left and right ventricular stroke volume using volumetric analyses of SSFP cine sequences to measure single valve regurgitation.

b. Mitral regurgitation can be measured by subtracting aortic flow from the LV stroke volume.

c. Multiple valve lesions can be assessed from comparison of the aortic and pulmonary diastolic regurgitant flow and the LV and RV stroke volumes.

d. Measure aortic valve area by direct planimetry

e. Alternatively, measure aortic valve area by (Velocity time integral LVOT/Velocity time integral aorta) × Area LVOT

f. Calculate peak mitral valve gradient from peak mitral valve flow

#### 2.3.5. Pericardial disease

1. LV structure and function module

2. T1 or T2-weighted fast spin echo images

a. 2–3 representative long axis images and 3–4 representative short axis images to measure pericardial thickness (normal ≤ 3 mm)

b. If pericardial cyst is suspected, refer to masses protocol

3. Optional – If regions of thickened pericardium noted – T1-weighted gradient echo myocardial tagged cine sequences to demonstrate presence or absence of epicardial/pericardial slippage (2–3 long axis images and 1–2 short axis images)

4. Optional, but encouraged if available – real-time imaging in the short axis during dynamic breathing maneuvers for evaluation of ventricular interdependence

5. Late Gadolinium Enhancement module

#### 2.3.6. Cardiac and paracardiac masses, including thrombi

1. LV structure and function module

2. T1 weighted fast spin echo – slices through the mass and surrounding structures (number of slices depends on size of the mass)

3. T2 weighted fast spin echo with fat suppression (optional – without fat suppression) – through the mass and surrounding structures as above. See nonischemic cardiomyopathies for sequence details.

4. First pass perfusion module with slices through mass

5. Repeat T1 weighted turbo spin echo with fat suppression

6. (optional) Repeat selected steady state free precession cine images post-contrast

7. Late gadolinium enhancement module – note that TI to null the mass may be different than for myocardium.

## Authors' contributions

CMK wrote protocols, edited protocols, edited manuscript, corresponding author, JB wrote protocols, edited protocols, edited manuscript, SDF wrote protocols, edited protocols, edited manuscript, RJK wrote protocols, edited protocols, edited manuscript, EN wrote protocols, edited protocols, edited manuscript. All authors read and approved the final manuscript.

